# Pure and stable metallic phase molybdenum disulfide nanosheets for hydrogen evolution reaction

**DOI:** 10.1038/ncomms10672

**Published:** 2016-02-10

**Authors:** Xiumei Geng, Weiwei Sun, Wei Wu, Benjamin Chen, Alaa Al-Hilo, Mourad Benamara, Hongli Zhu, Fumiya Watanabe, Jingbiao Cui, Tar-pin Chen

**Affiliations:** 1 Department of Physics and Astronomy, University of Arkansas at Little Rock, 2801 South University Avenue, Little Rock, Arkansas 72204, USA; 2 Department of Physics, University at Buffalo, Buffalo, New York 14260, USA; 3 Institute for Nanoscale Materials Science and Engineering, University of Arkansas, Fayetteville, Arkansas 72701, USA; 4 Department of Mechanical and Industrial Engineering, Northeastern University, Boston, Massachusetts 02115, USA; 5 Department of Physics and Materials Science, University of Memphis, Memphis, Tennessee 38152, USA

## Abstract

Metallic-phase MoS_2_ (M-MoS_2_) is metastable and does not exist in nature. Pure and stable M-MoS_2_ has not been previously prepared by chemical synthesis, to the best of our knowledge. Here we report a hydrothermal process for synthesizing stable two-dimensional M-MoS_2_ nanosheets in water. The metal–metal Raman stretching mode at 146 cm^−1^ in the M-MoS_2_ structure, as predicted by theoretical calculations, is experimentally observed. The stability of the M-MoS_2_ is associated with the adsorption of a monolayer of water molecules on both sides of the nanosheets, which reduce restacking and prevent aggregation in water. The obtained M-MoS_2_ exhibits excellent stability in water and superior activity for the hydrogen evolution reaction, with a current density of 10 mA cm^−2^ at a low potential of −175 mV and a Tafel slope of 41 mV per decade.

## Abstract (web summary)

Metallic molybdenum disulfide is a metastable phase of the material. Here, the authors synthesize two-dimensional metallic molybdenum disulfide nanosheets, stabilized by adsorbed aqueous monolayers, and evaluate their catalytic hydrogen evolution activity.

## Graphical abstract

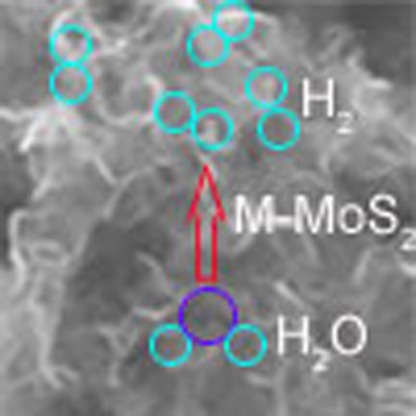

The metallic-phase MoS_2_ (M-MoS_2_) is a single-layer S-Mo-S structure in which each molybdenum atom is surrounded by six sulfur atoms in an octahedral lattice. With dense active sites and an electronic conductivity that is six orders of magnitude higher than that of the semiconductor phase of MoS_2_ (S-MoS_2_) (ref. 1), M-MoS_2_ has emerged as a promising candidate for a broad range of applications and is expected to exhibit better performance than its semiconducting counterpart, in particular in the hydrogen evolution reaction (HER) and as a photocatalyst and supercapacitor^2^,^3^,^4^,^5^. These attractive properties of M-MoS_2_ are offset by the fact that a pure phase of M-MoS_2_ is very challenging to prepare, because it is highly unstable. Over the past few decades, many efforts to develop a process to prepare stable and highly pure M-MoS_2_ have largely proved futile. A complicated method for the preparation of metastable M-MoS_2_ using lithium intercalation of S-MoS_2_ has been reported^5^,^6^,^7^,^8^. However, the reported M-MoS_2_ suffered from coexistence with S-MoS_2_ in a relative proportion of 50–80% (refs 3, 9). In addition, the intermediary Li_*x*_MoS_2_ and intercalator of n-butyllithium are both dangerous materials that may self-heat^8^ and are highly pyrophoric in air^5^. The preparation procedures are also tedious and can take as long as 3 days^10^,^11^. The previously mentioned M-MoS_2_ easily transformed to S-MoS_2_ due to S–S van der Waals interactions^12^,^13^,^14^. Therefore, an efficient synthesis method for the production of a large quantity of stable and pure M-MoS_2_ is still highly desirable.

M-MoS_2_ could be an intermediary state during the synthesis of S-MoS_2_, which is a stable form in many chemical reactions^15^,^16^. This approach provides a possible method for capturing the metastable M-MoS_2_ even though it is thermodynamically unstable and may rapidly transform to S-MoS_2_ at elevated temperatures. In fact, S-MoS_2_ can transform into an octahedral structure of M-MoS_2_ under a high pressure of 35 GPa (refs 17, 18, 19), which implies that the metallic phase of MoS_2_ may be achievable at a relatively low temperature, a specific pressure and a proper chemical environment. Therefore, we employ octahedral MoO_3_ as the starting material in a pressurized hydrothermal process, to directly grow highly pure M-MoS_2_ in water. The as-prepared M-MoS_2_ is highly stable in water due to the reduced stacking of the layered materials, which are separated by water molecules. Urea, which is a weak reducing agent, plays a key role in the formation of M-MoS_2_. The structure of octahedral MoO_3_ can be maintained under acidic conditions. To satisfy the expected growth conditions, thioacetamide is chosen as the sulfur source and the reaction is maintained at a low pH value (pH∼4). The synthesis of M-MoS_2_ is performed at 200 °C by mixing MoO_3_, thioacetamide and urea in an autoclave. The as-prepared M-MoS_2_ is pure and stable with high activity for the HER.

## Results

### Highly pure metallic phase MoS_2_

**Figure 1:**
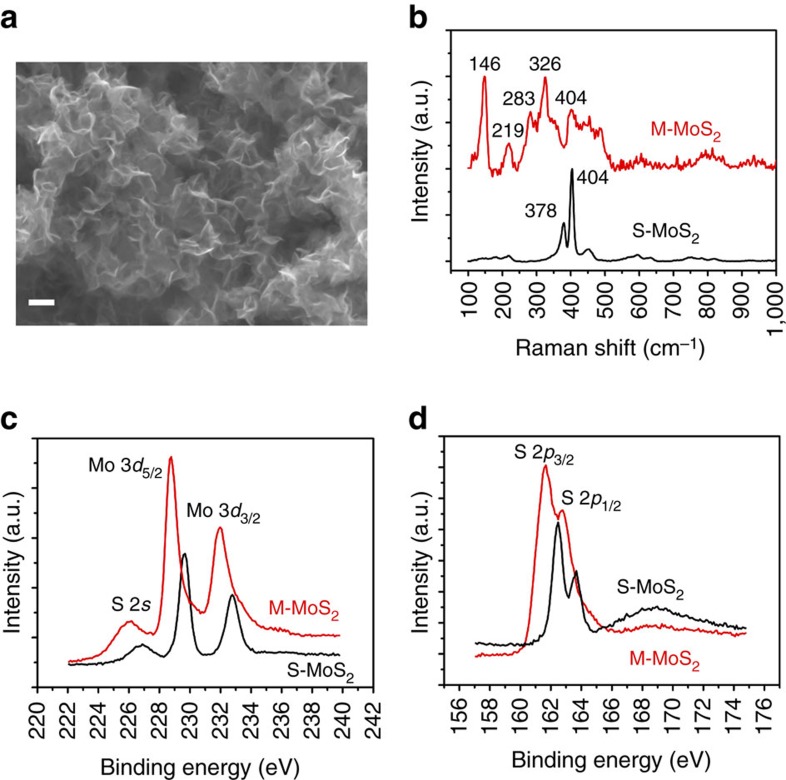
Morphology of M-MoS_2_ and phase identification of M-MoS_2_ and S-MoS_2_. (**a**) SEM image of M-MoS_2_ showing the nanosheet structures. Scale bar, 100 nm. (**b**) Raman shift of M-MoS_2_ and S-MoS_2_. (**c**,**d**) High-resolution X-ray photoelectron spectroscopy (XPS) spectra of Mo 3*d* (**c**) and S 2*p* (**d**) for M-MoS_2_ and S-MoS_2_.

Figure 1a shows a typical scanning electron microscopic (SEM) image of the as-prepared M-MoS_2_ sample. The MoS_2_ nanosheets are 100 nm in size and a few nanometres in thickness. The morphology of S-MoS_2_ is similar to that of M-MoS_2_ on the nanoscale (Supplementary Fig. 1). The results from energy dispersive X-ray spectroscopy indicated that the S to Mo atomic ratio of the as-prepared M-MoS_2_ is ∼2.05 (Supplementary Fig. 2). The most notable difference between M-MoS_2_ and S-MoS_2_ is the symmetry of the sulfur in their structures. The structural variations result in significant differences in their characteristic Raman features. Figure 1b shows the Raman spectra of M-MoS_2_ and S-MoS_2_ synthesized at 200 °C and 240 °C, respectively. A strong Raman band is observed at 146 cm^−1^ and attributed to Mo–Mo stretching vibrations in M-MoS_2_. Calculations have predicted this band at the *K* points of the Brillouin zone for octahedrally coordinated distorted MoS_2_ (ref. 12). Our results confirm the existence of this band in M-MoS_2_. The Raman shifts (that is, ∼219, 283 and 326 cm^−1^) are also associated with the phonon modes in M-MoS_2_. S-MoS_2_ exhibits typical Raman shifts of ∼378 and 404 cm^−1^ for E^1^_2g_ and A_1g_, respectively, which are substantially different from those of M-MoS_2_.

The phase identification of the synthesized M-MoS_2_ and S-MoS_2_ was further studied using X-ray photoelectron spectroscopy and the results are shown in Fig. 1c,d. The Mo 3*d* spectra consist of peaks located at approximately 228.7 and 231.8 eV, which correspond to the 3d_5/2_ and 3*d*_3/2_ components, respectively, of Mo^4+^ in M-MoS_2_. However, the Mo 3*d* peaks of the M-MoS_2_ samples also exhibit weak shoulders at the positions as those in S-MoS_2_ due to the transformation of M-MoS_2_ in vacuum and X-ray illumination. The details for the peak deconvolution are provided in Supplementary Fig. 3 and Supplementary Note 1. The Mo 3*d* peaks of M-MoS_2_ shifted to lower binding energies by ∼1 eV with respect to the corresponding peaks in S-MoS_2_. This result is consistent with the previously obtained relaxation energy of 1 eV for M-MoS_2_ derived from S-MoS_2_ (refs 20, 21). Similarly, the S 2*p* peaks of M-MoS_2_ were located at ∼161.6 and 162.7 eV, and associated with S 2*p*_3/2_ and 2*p*_1/2_, respectively, which are also ∼1 eV lower than the corresponding peaks in S-MoS_2_. It is important to note that the prominent S peak located at ∼168 eV was absent, indicating that the sulfur atoms remain unoxidized in M-MoS_2_. However, this peak was observed in S-MoS_2_ after annealing at 240 °C.

### Morphologies and optical properties of MoS_2_

**Figure 2:**
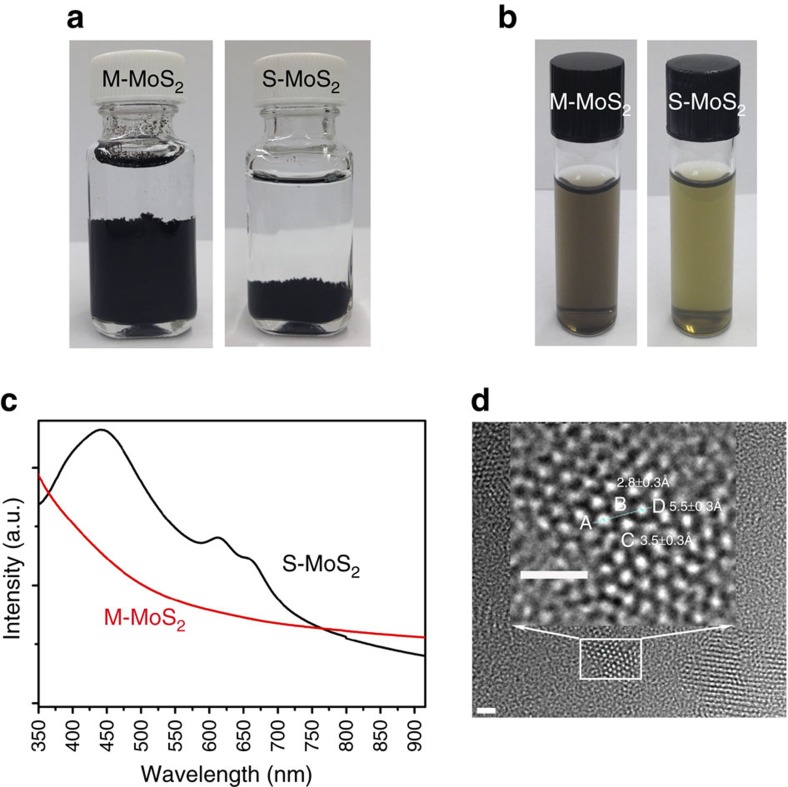
Morphology and property of M-MoS_2_ and S-MoS_2_. (**a**) As-prepared M-MoS_2_ and S-MoS_2_ in water. (**b**) Suspension of M-MoS_2_ and S-MoS_2_ in water. (**c**) Ultraviolet–visible absorption of M-MoS_2_ and S-MoS_2_. (**d**) HRTEM image of M-MoS_2_. The region indicated by the squares is enlarged to show the atomic structure of M-MoS_2_. Scale bar, 1 nm.

Figure 2a shows optical images of the two types of MoS_2_ dispersed in water. M-MoS_2_ does not appear to exhibit bulk aggregation, whereas S-MoS_2_ aggregates at the bottom of the solution. This result may be because M-MoS_2_ possesses hydrophilic surfaces, whereas S-MoS_2_ possesses relatively hydrophobic surfaces^5^,^22^. After sonication, both M-MoS_2_ and S-MoS_2_ can uniformly disperse in water (Fig. 2b). However, M-MoS_2_ was grey colour, whereas S-MoS_2_ was green colour. These colours were associated with their optical properties, which were confirmed by optical absorption spectroscopy. Figure 2c shows the ultraviolet–visible absorption spectra of M-MoS_2_ and S-MoS_2_ in water. Two typical absorption peaks located at 613 and 660 nm were observed for S-MoS_2_ and these peaks are associated with the energy split from the valence band spin–orbital coupling in S-MoS_2_ with large lateral dimensions^23^. In addition, another optical absorption band located at ∼442 nm was observed. This band most probably results from the quantum effect of smaller lateral-sized S-MoS_2_ nanosheets. The absorption spectrum of M-MoS_2_ has no salient absorption bands but a monotonic change that is indicative of its metallic property.

The atomic structure of M-MoS_2_ was investigated using a high-resolution scanning transmission electron microscope (HRTEM) at different magnifications (Supplementary Fig. 4) and the high-magnification image is shown in Fig. 2d. The hexagonal atomic arrangement of Mo indicates that each individual nanosheet consists of single crystal domains. Mo atoms on the basal plane have a Mo–Mo distance from *A* to *D* of 5.5±0.3 Å, *A* to *B* of 2.8±0.3 Å and *A* to *C* of 3.5±0.3 Å. However, the crystal lattice may convert into an amorphous structure under the high-energy electron beam of TEM as observed by Lukowski *et al*.^2^.

### Stability of the M-MoS_2_

**Figure 3:**
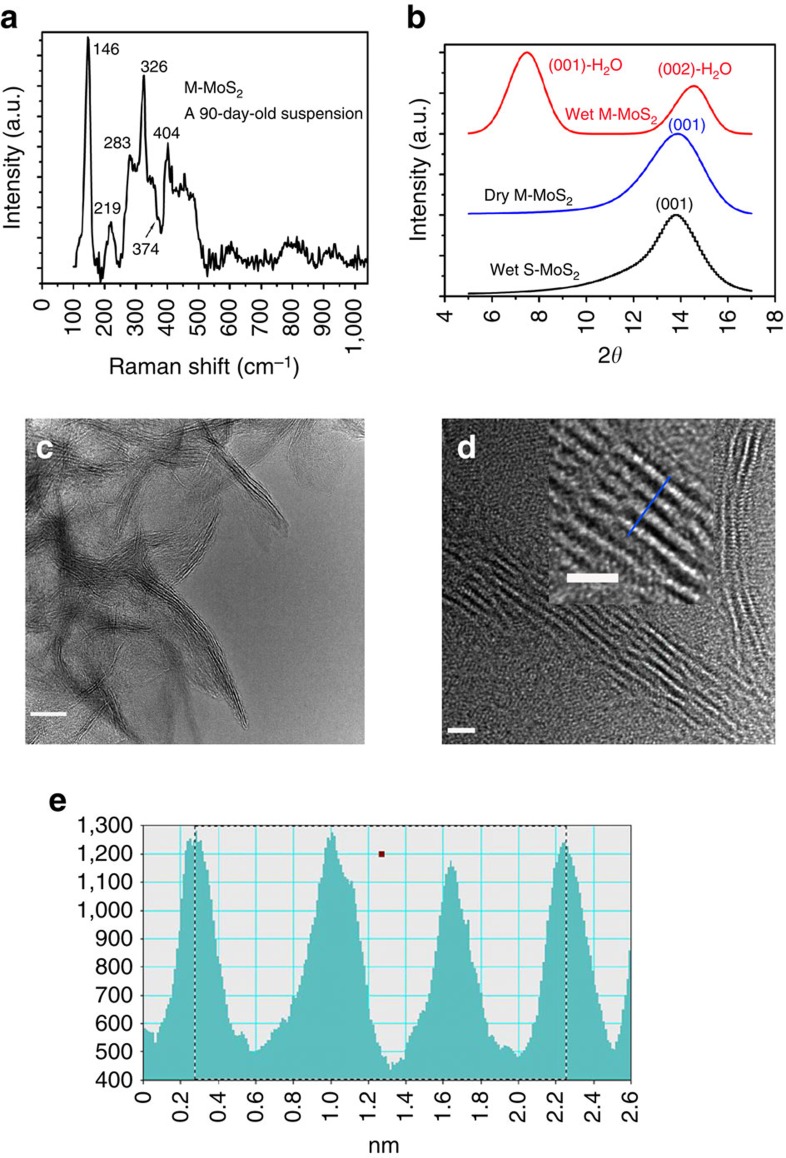
Raman and structural characterization to investigate the stability of M-MoS_2_. (**a**) Raman shift of M-MoS_2_ stored for 90 days at room temperature. (**b**) XRD patterns of wet M-MoS_2_, dry M-MoS_2_ and wet S-MoS_2_. (**c**) Low-resolution TEM image of M-MoS_2_. Scale bar, 10 nm. (**d**) HRTEM image of M-MoS_2_. The region indicated by the square is enlarged to show the layered structure of M-MoS_2_. Scale bar, 2 nm. (**e**) Line scan of the HRTEM image indicated by the blue line in **d**, indicating a layer-to-layer spacing of 0.65 nm.

The as-prepared M-MoS_2_ nanosheets in water exhibited good stability over time. We believe that the high purity of M-MoS_2_ plays an important role in its stability. 1T-MoS_2_ (metallic phase), which was derived from 2H-MoS_2_ (semiconductor phase), consists of electronic heterostructures of 2H-MoS_2_ and 1T-MoS_2_ in individual layers of MoS_2_ (ref. 9). The coexistence of semiconductor MoS_2_ with a metallic phase in a single layer resulted in an expedited transformation of 1T back to 2H MoS_2_. Therefore, the structure was reported to be stable for 12 days with the observation of the 2H structure in water for chemically exfoliated MoS_2_ (ref. 12). The samples obtained in this study were stored in water for more than 90 days at room temperature and no obvious change in the Raman shift was observed (Fig. 3a). The characteristic peaks of the stored M-MoS_2_ were similar to those observed for the freshly prepared samples. This observation indicates that the as-prepared M-MoS_2_ was highly stable in water. It is important to note that a traceable transformation of M-MoS_2_ to S-MoS_2_ may occur, as suggested by the very weak shoulder at 374 cm^−1^. The pure metallic phase in the individual layers contributes to the stability of the material. A comparison of the Raman spectra of M-MoS_2_ stored under different conditions and S-MoS_2_ is shown in Supplementary Fig. 5.

Another possible stability mechanism may arise from the monolayer of water molecules that was adsorbed on both sides of the M-MoS_2_ nanosheets. This layer prevents aggregation and stacking, and maintains the octahedral structure in M-MoS_2_. The existence of the water molecule layer was confirmed by XRD measurements (Fig. 3b). The peak at 7.5°, which corresponds to a spacing of 11.8 Å, was identified as (001)-H_2_O for M-MoS_2_-H_2_O, which contains a bilayer of water molecules between the adjacent layers of MoS_2_ nanosheets^24^. As the layer thickness of MoS_2_ was ∼6.2 Å and the thickness of a monolayer of water was 2.8 Å, the total thickness of a MoS_2_ layer surrounded by a water bilayer would be 11.8 Å. This result was previously confirmed by XRD^25^. The peak located at 14.6° was assigned to the second-order reflection of M-MoS_2_-H_2_O.

XRD was also performed on the M-MoS_2_ nanosheets after they were dried for 24 h in a vacuum chamber at a pressure of 6 mTorr. The XRD pattern of the dried M-MoS_2_ is similar to that of S-MoS_2_ with a peak at 13.88° (Fig. 3b), which corresponds to the stacked M-MoS_2_ multilayers. This result suggests that the spacing between the Mo planes for M-MoS_2_ was 6.4±0.1 Å. This spacing is larger than that of bulk MoS_2_ (that is, 6.15 Å). The diffraction peak at 7.5° disappeared due to desorption of the water molecules on the nanosheet surfaces. The TEM and HRTEM images indicate that M-MoS_2_ was composed of single-layer or several-layer structures (Fig. 3c,d). The lattice fringe spacing of M-MoS_2_ was measured to be 1.97 nm for a four-layer nanosheet, which produced a Mo–Mo spacing of 6.5±0.1 Å (Fig. 3e). This result is consistent with the value obtained from the XRD measurements. The increased interlayer spacing in M-MoS_2_ may contribute to its stability due to the weakened S–S van der Waals interactions and reduced restacking probability.

M-MoS_2_ contained adsorbed water molecules, which was confirmed by XRD measurements. However, this phenomenon was not observed in S-MoS_2_ nanostructures. This difference between the two phases of MoS_2_ reflects the different surface properties (that is, M-MoS_2_ is hydrophilic, whereas S-MoS_2_ is hydrophobic). These properties are determined by the atomic structures of M-MoS_2_ and S-MoS_2_. The Mo atom in M-MoS_2_ is located in an octahedral S coordination, whereas the Mo atom in S-MoS_2_ coordinated by six S atoms in a trigonal prismatic arrangement with S acting as a hydrophobic site and Mo serving as a hydrophilic site^26^. These different surface properties were also observed regardless of whether they were dispersed in water or deposited as thin films on substrates (Supplementary Fig. 6). Contact angle measurements were conducted on both the M-MoS_2_ and S-MoS_2_ films. A contact angle of 25° was observed for M-MoS_2_ and an angle of 118° was determined for S-MoS_2_, confirming the highly hydrophilic surface of M-MoS_2_. The details are provided in Supplementary Fig. 7.

### Metallic phase MoS_2_ for HER

**Figure 4:**
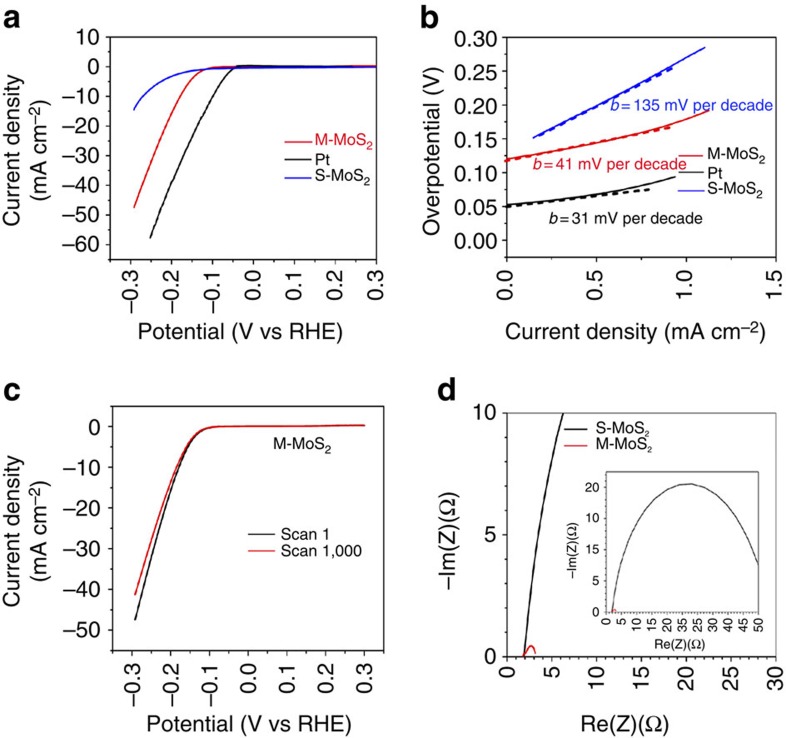
HER activity of the synthesized MoS_2_ nanosheets. (**a**) Polarization curves of the M-MoS_2_ and S-MoS_2_ nanosheets. (**b**) Corresponding Tafel plots obtained from the polarization curves. The Tafel slopes were ∼41 and ∼135 mV per decade for M-MoS_2_ and S-MoS_2_, respectively. (**c**) Polarization curves of the M-MoS_2_ nanosheets after 1 and 1,000 cycles of continuous operation. (**d**) Nyquist plots of M-MoS_2_ and S-MoS_2_.

The different surface properties, atomic structure and morphology of M-MoS_2_ and S-MoS_2_ on substrates led to significant differences in the HER. Hydrogen production measurements were conducted for the M-MoS_2_ and S-MoS_2_ nanosheets deposited on glassy carbon electrodes using a three-electrode cell in a 0.5-M sulfuric acid electrolyte. Figure 4a,b show the polarization curves and corresponding Tafel plots of the M-MoS_2_ and S-MoS_2_ nanosheets compared with those of Pt. It is important to note that the Tafel plot employs a logarithmic current density axis. A low potential of −175 mV at a current density of 10 mA cm^−2^ and a Tafel slope of 41 mV per decade were obtained for M-MoS_2_. This observed potential for hydrogen production represents the lowest value among the reported experimental data for the use of only MoS_2_ materials. The long-term stability of the M-MoS_2_ electrode for the HER was investigated. After 1,000 cycles of continuous operation, the exfoliated MoS_2_ nanosheets exhibited <12% decay in the electrocatalytic current density (Fig. 4c). The advantage of using M-MoS_2_ nanosheets becomes more apparent through electrochemical impedance spectroscopy analysis. The electrochemical impedance spectroscopy was performed under the HER conditions. The Nyquist plots are shown in Fig. 4d and revealed a dramatically decreased charge transfer resistance for the M-MoS_2_ nanosheets (∼1 Ω) relative to the S-MoS_2_ nanosheets (∼50 Ω). Furthermore, we measured the resistivity of the M-MoS_2_ (300 nm thick) and S-MoS_2_ (340 nm thick) films using a four-probe method under ambient conditions. The sheet resistances are 1.61 × 10^4^ Ω/□ for M-MoS_2_ and 2.26 × 10^9^ Ω/□ for S-MoS_2_, which correspond to a resistivity of 0.482 Ω·cm for M-MoS_2_ and 7.68 × 10^4^ Ω·cm for S-MoS_2_. This result indicates that M-MoS_2_ has a conductivity that is five orders of magnitude higher than that of S-MoS_2_.

It is important to note that the growth of M-MoS_2_ and the HER measurements are highly reproducible. The growth process of M-MoS_2_ failed only once in 96 trials. The failure was caused by washing the autoclave with HNO_3_ and we believe that a trace amount of residual HNO_3_ may have oxidized the products via different chemical reactions. The autoclave was typically cleaned by sonication in water. We have performed the HER 62 times using our synthesized M-MoS_2_ and all of the trials yielded results similar to those shown in Fig. 4a,b. The exceptional performance of M-MoS_2_ for hydrogen production may be due to the high purity, dense active sites, good conductivity and excellent hydrophilic property of M-MoS_2_. By contrast, S-MoS_2_ exhibited little HER activity with a potential of −274 mV at 10 mA cm^−2^ and a Tafel slope of 135 mV per decade. The poor performance of S-MoS_2_ in the HER was due to few active sites, aggregation and its hydrophobic surface. The exchange current density of M-MoS_2_, S-MoS_2_ and Pt is discussed in Supplementary Discussion. Although M-MoS_2_ still lags behind Pt, improvement in the HER activity can be accomplished by enhancing the conductivity of the nanoscale M-MoS_2_. Therefore, metallic MoS_2_ exhibits great potential for use as a replacement for Pt in the HER as a low-cost and highly active catalyst for practical applications.

## Discussion

The mechanism underlying the formation of the stable metallic phase of MoS_2_ is directly associated with the weak reducer urea, which can precisely and effectively reduce MoO_3_ to M-MoS_2_ as shown in equation (1). The absence of a urea reducer while maintaining other growth conditions would result in a product dominated by amorphous MoS_3_, as shown in equation (2) (see Supplementary Methods and Supplementary Fig. 8). Other reducers, such as hydrazine, reduce MoO_3_ to S-MoS_2_ only due to the relatively strong electron donating ability of hydrazine.


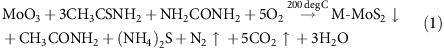



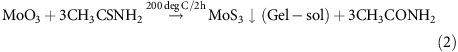



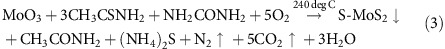


During the synthesis of M-MoS_2_, the three most important factors are solvent, pH and temperature. First, water serves as a solvent and is adsorbed on the surface of M-MoS_2_ to form a water molecular layer that prevents layer stacking. When an organic solvent, such as ethanol or dimethylformamide, was added to the reaction, no desirable product was produced (see the Supplementary Methods and Supplementary Fig. 9). No solid product was found in the autoclave when a 1:1 ratio of water to ethanol was used. Second, the pH controls the steric structure of the starting materials and the formation of M-MoS_2_. When the pH value was ≤4, the octahedral coordination of MoO_3_ can be maintained, which may lead to the formation of M-MoS_2_. At higher pH values (pH 8) achieved through the addition of ammonia, the MoO_3_ structure converts into MoO_4_^2−^ tetrahedral centres. This change causes the reaction to yield no desirable products. Third, the temperature also plays a key role in the formation of M-MoS_2_. In this study, pure M-MoS_2_ was obtained at 200 °C and S-MoS_2_ was formed at 240 °C in the reaction listed in equation (3).

**Figure 5:**
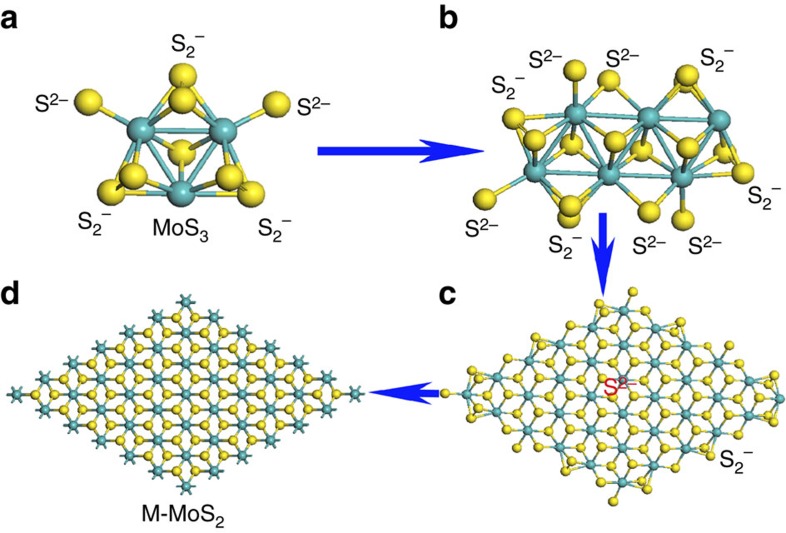
Schematic illustrations of the evolution process of M-MoS_2_ from MoS_3_. (**a**) One MoS_3_ cluster with S^2−^ and S_2_^−^. (**b**) Combination of two MoS_3_ clusters with the breaking of S_2_^−^. (**c**) Several MoS_3_ cluster forming MoS_2_ with S^2−^ in the centre and MoS_3_ with S_2_^−^ at the edge. (**d**) Stable M-MoS_2_ crystal structure.

Based on these discussions, the metastable M-MoS_2_ intermediate can be formed and stabilized in a pressurized hydrothermal process with a suitable reducer and temperature. The proposed mechanism involves the formation of a crystal phase of M-MoS_2_ from amorphous clustered MoS_3_ with S_2_^−^ edge bonds reduced by urea. In fact, many investigations have revealed the structural evolution from MoS_3_ to S-MoS_2_ without the MoS_2_ metallic phase during the evolution process^27^,^28^,^29^. We believe that the high temperature and strong reducer used in the previous studies may have only resulted in the formation of semiconductor MoS_2_. This result was also confirmed in this study, because only S-MoS_2_ was obtained when the temperature was increased to 240 °C. The evolution process for the formation of M-MoS_2_ is shown in Fig. 5, which illustrates the growth of MoS_3_ from one cluster to two and finally to a nanosheet. The central part possesses the structure of M-MoS_2_ and the S_2_^−^ bond on the edge can be removed by urea to continue the growth to form a single layer of M-MoS_2_ (ref. 30). A control experiment was carried out by stopping the growth after 90 min, which resulted in a mixture of MoS_3_ and M-MoS_2_ in the solution. The existence of MoS_3_ and its corresponding peak at 120 cm^−1^ peak was confirmed by Raman spectroscopy^31^. Furthermore, a Raman band located at 548 cm^−1^, which was due to the S_2_^−^ stretching vibration in MoS_3_, was observed. Energy dispersive X-ray spectroscopy measurements indicate a ratio of 1:3 for Mo and S (See Supplementary Fig. 10b). The Mo–Mo vibration mode at 146 cm^−1^ starts to appear and become more prominent as growth time increased, indicating the transformation of MoS_3_ to M-MoS_2_ (see Supplementary Fig. 10c). After continuing the reaction for 6 h, only M-MoS_2_ was observed in the product. At this point, the Raman peak located at 120 cm^−1^ disappeared and the intensity of the 146 cm^−1^ peak reached its maximum. This result indicates the complete conversion of MoS_3_ to M-MoS_2_.

A hydrothermal process has been used to grow S-MoS_2_. To the best of our knowledge, the preparation of M-MoS_2_ using a hydrothermal method has not been previously reported. We believe that the growth conditions used in this study play a key role in the formation and stabilization of M-MoS_2_. M-MoS_2_ is an unstable phase that can easily transform to S-MoS_2_. Therefore, the octahedral coordination of M-MoS_2_ must be maintained during deposition. For this reason, we used MoO_3_ as the starting material, because it possesses the same octahedral structure as M-MoS_2_. The metallic phase can be obtained when the crystal structure can be maintained during the conversion process from MoO_3_ to MoS_2_. Urea, which is a weak reducer, is able to maintain the crystal structure at a low pH value and optimum temperature (200 °C) for hydrothermal growth. As mentioned in the previous sections, only S-MoS_2_ was obtained at a higher growth temperature of 240 °C, because the octahedral structure of M-MoS_2_ cannot be maintained under the growth conditions used. Once the M-MoS_2_ nanosheets were formed at 200 °C, the adsorption of water molecules on their surfaces due to the hydrophilic nature of the surface provides an environment to maintain the octahedral structure and preventing restacking of the layers. Therefore, M-MoS_2_ can be stored in water for several months without significant transition to S-MoS_2_. However, once M-MoS_2_ is removed from water and dried in a vacuum, it will slowly convert to S-MoS_2_. This observation indicates that the stability of M-MoS_2_ is dependent on the environment. Although this study was focused on the influence of water, the environmental stability of M-MoS_2_ under other conditions, such as different gases and solutions other than water, should be considered in future investigations.

In conclusion, highly pure and stable M-MoS_2_ nanosheets were synthesized in solution using a rational and controllable approach. A unique Raman peak located at 146 cm^−1^ due to M-MoS_2_ was experimentally observed, which confirms the theoretical prediction. A growth mechanism was proposed and is supported by the experimental data. Excellent activity of M-MoS_2_ for the HER was achieved and the material has the potential to replace Pt in practical applications. These results provide opportunities for studying the metallic phase of MoS_2_, which has potential technological applications in renewable energy, energy storage and heterogeneous catalysis.

## Methods

### Preparation of M-MoS_2_ and S-MoS_2_

MoO_3_ powder (CAS number is 1311-27-5) and urea (CAS number is 57-13-6) were purchased from the Sigma-Aldrich Company. Thioacetamide (CAS number is 62-55-5) was purchased from the Fisher Scientific Company. The autoclave (model number 4749) was ordered from the Parr Instrument Company.

Twelve milligrams of MoO_3_, 14 mg of thioacetamide and 0.12 g of urea were dissolved in 10 ml of deionized water and stirred for 2 h. Then, the solution was placed in an autoclave and loaded into a furnace (from MTI), which has been heated to 200 °C. The temperature of the oven was maintained at 200 °C for 12 h. Next, the reaction was terminated by rapidly cooling the solution to room temperature by removing the autoclave from the oven. The prepared M-MoS_2_ was collected and washed with deionized water several times, followed by storage in deionized water before use. The same procedure was used to synthesize S-MoS_2_ at 240 °C. Two-dimensional nanosheets were observed and no significant difference was observed between M-Mo_2_ and S-MoS_2_ based on their nanoscale morphology.

### Ultraviolet–visible–infrared spectroscopy

Ultraviolet–visible–infrared absorption spectroscopy of the M-MoS_2_ and S-MoS_2_ dispersions in water were recorded using a Perkin Elmer Lambda 25 spectrophotometer.

### TEM observations

TEM and HRTEM images and electron diffraction were performed on a FEI Tecnai G2 F20 S-Twin microscope operated at an accelerating voltage of up to 200 kV. The TEM samples were prepared by sonication at 500 W for ∼5 min and 25 μl of the supernatant were dropped onto holey carbon grids.

### SEM observations

The SEM images were obtained using a field-emission gun SEM (Quanta 400 FEG FEI). The samples were dispersed in water and then dropped onto the Si/SiO_2_ substrates.

### X-ray photoelectron spectroscopy

X-ray photoelectron spectroscopy was conducted using an Axis Ultra DLD (Kratos) system. The vacuum of the chamber was 1 × 10^−9^ Torr. A monochromatic aluminum *K*_α_ source with a source power of 150 W (15 kV × 10 mA) was used. The pass energy was 160 eV for wide scans and 40 eV for narrow scans.

### Raman spectroscopy

Raman spectroscopy was performed using a LabRam HR800 UV NIR and 532-nm laser excitation with working distances on a × 50 lens. The Raman spectra of M-MoS_2_ and S-MoS_2_ were recorded by depositing the samples on silicon substrates.

### XRD patterns

The XRD patterns of M-MoS_2_ and S-MoS_2_ were recorded for two theta values ranging from 4° to 18°, to characterize the interlayer spacing. The characterization was performed on a Bruker AXS-D8 Advance powder X-ray diffractometer using Cu/Ka radiation (*λ*=1.5406 Å) with a step size of 0.02° and a dwell time of 3.0 s.

### Electrocatalytic hydrogen evolution

The electrochemical measurements were performed using a three-electrode electrochemical station (Gamry Instruments). All of the measurements were performed in a solution consisting of 50 ml of a 0.5-M H_2_SO_4_ electrolyte prepared using 18 M deionized water purged with H_2_ gas (99.999%). The MoS_2_ suspension in water was dropped onto glassy carbon as the working electrode. All of the working electrodes were prepared using the MoS_2_ solutions. The solution concentrations of M-MoS_2_ and S-MoS_2_ were ∼0.3 mg ml^−1^ and ∼0.4 mg ml^−1^, respectively, in water. The diameter of the glass carbon electrode was 0.3 cm and the area was ∼0.07 cm^2^. The amount of deposited catalyst on the electrode was ∼ 43 μg cm^−2^ for M-MoS_2_ and 114 μg cm^−2^ for S-MoS_2_. Platinum foil was used as the counter electrode and a saturated calomel was used as the reference electrode. The reversible hydrogen electrode was calibrated using platinum as both the working and counter electrodes to +0.3 V versus the saturated calomel reference electrode. The performance of the hydrogen evolution catalyst was measured using linear sweep voltammetry from +0.3 to −0.3 V versus reversible hydrogen electrode with a scan rate of 5 mV s^−1^.

## Additional information

**How to cite this article**: Geng, X. *et al*. Pure and stable metallic phase molybdenum disulfide nanosheets for hydrogen evolution reaction. *Nat. Commun.* 7:10672 doi: 10.1038/ncomms10672 (2016).

## Supplementary Material

Supplementary Information Supplementary Figures 1-10, Supplementary Note 1, Supplementary Discussion and Supplementary Methods
