# Supplementary material for: Pure and stable metallic phase molybdenum disulfide nanosheets for hydrogen evolution reaction
Source: Nat Commun. 2016 Feb 10;7:10672. doi: 10.1038/ncomms10672 (PMC4749985; doi:10.1038/ncomms10672)
Supplement: Supplementary Information — Supplementary Figures 1-10, Supplementary Note 1, Supplementary Discussion and Supplementary Methods [file ncomms10672-s1.pdf]

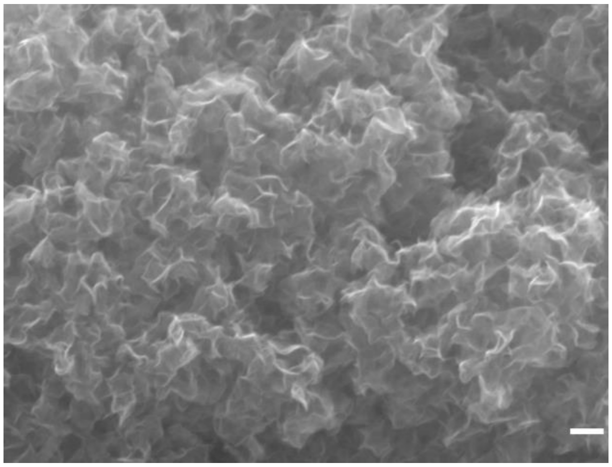

Supplementary Figure 1: SEM image of S-MoS_2. Scale bar, 100 nm. The morphology of S-MoS_2 is similar to that of M-MoS_2 at the nanoscale as shown by SEM images at high magnification.

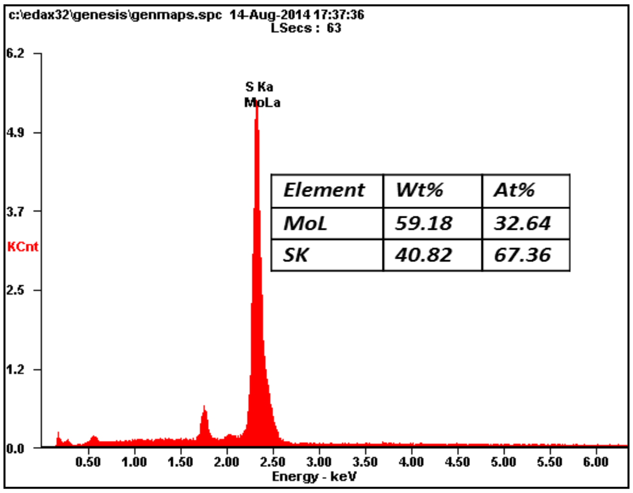

Supplementary Figure 2: EDX of M-MoS_2. The as-prepared M-MoS_2 was characterized by energy dispersive x-ray spectroscopy (EDX). The atomic ratio of S to Mo was calculated to be around 2.05.

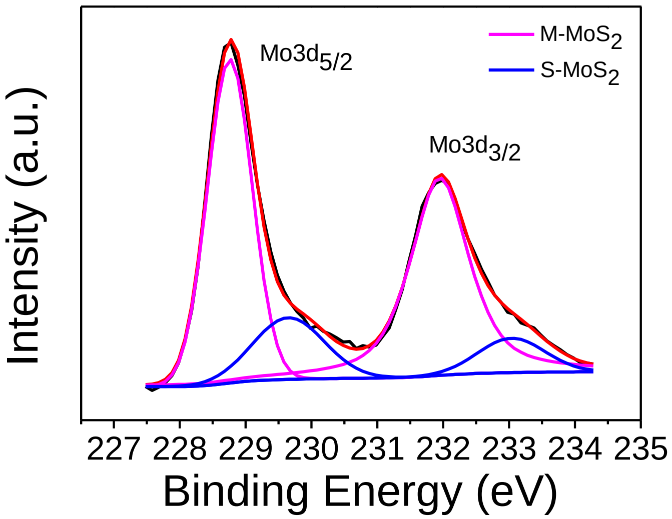

Supplementary Figure 3: XPS of M-MoS$_2$. After Shirley background subtraction, the Mo 3d peaks were de-convoluted to show the M-MoS$_2$ (pink) and S-MoS$_2$ (blue) contributions.

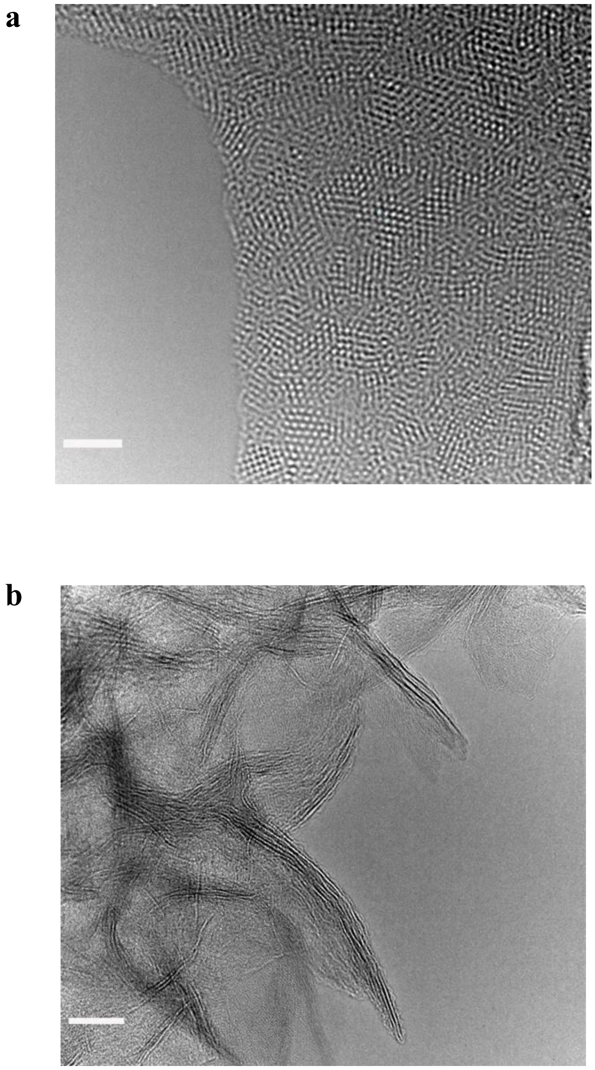

Supplementary Figure 4: TEM images M-MoS_2 nanosheets. (a) High magnification. Scale bar, 2 nm. (b) Low magnification. Scale bar, 10 nm.

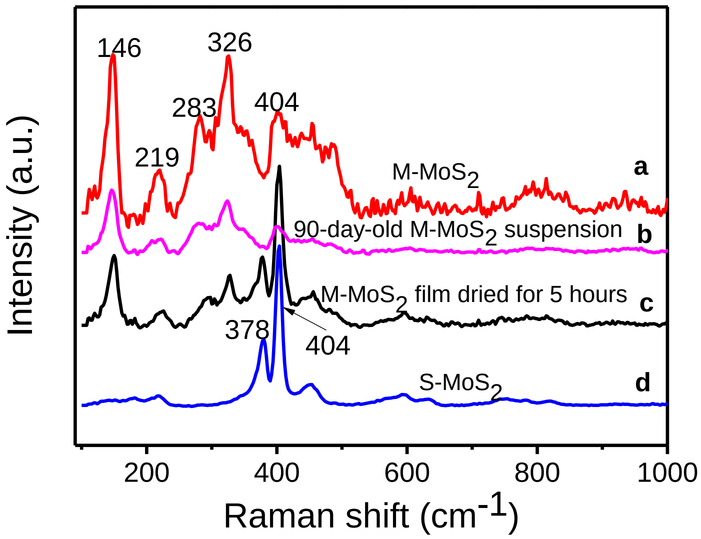

Supplementary Figure 5: Raman spectroscopy of different samples. (a) Fresh M-MoS_2 (Red). (b) 90-day-old M-MoS_2 stored in water (Pink), The M-MoS_2 sample stored for 90 days in water did not show obvious difference from freshly made M-MoS_2, indicating that the M-MoS_2 nanosheets are very stable in water. (c) M-MoS_2 film (Black). The M-MoS_2 films after drying for 5 hours in vacuum show an obvious peak at 378 cm^{-1}, which suggests that a fraction of M-MoS_2 has changed into S-MoS_2. (d) S-MoS_2 (Blue). Though the defects developed in air may affect the Raman features in terms of peak position and width, they would not affect the conclusion drawn from the Raman whether or not the material is metallic.

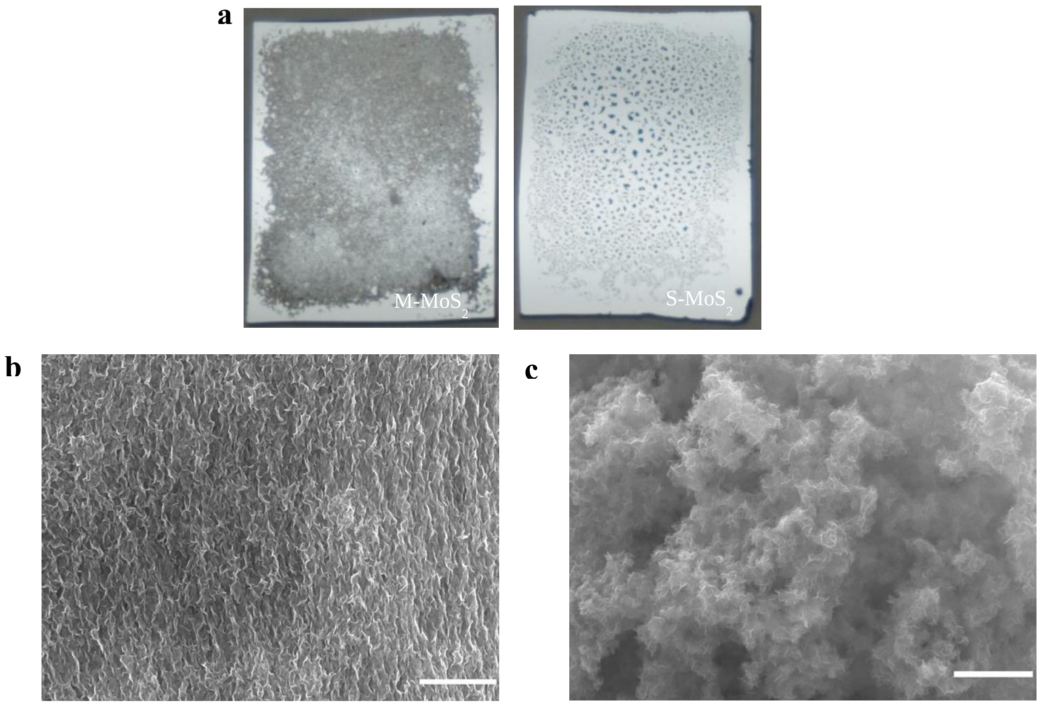

Supplementary Figure 6: Film morphologies of M-MoS_2 and S-MoS_2. (a) Optical images of the M-MoS_2 and S-MoS_2 on silicon substrates. (b and c) SEM images of M-MoS_2 and S-MoS_2 films. Scale bar, 1 µm.

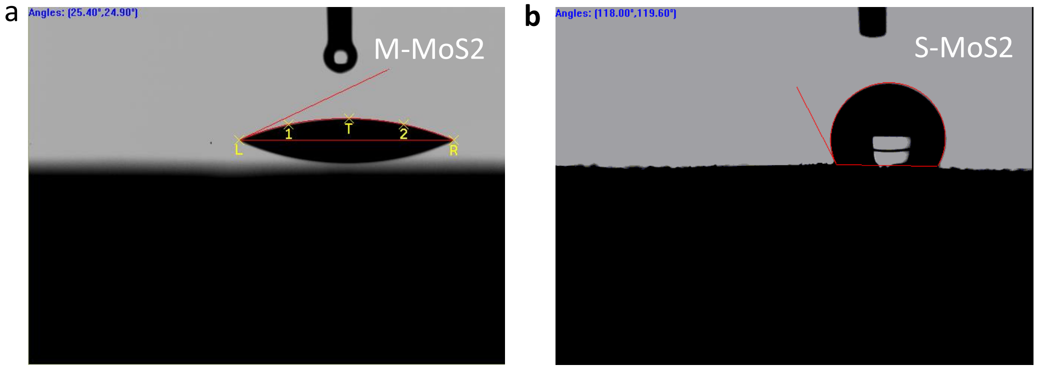

Supplementary Figure 7: Static contact angle images. (a) Fresh M-MoS_2 film. We measured the contact angle by drop casting a droplet of water (5 μL) on the M-MoS_2 film and obtained an angle of 25 °. (b) S-MoS_2 film. The same experiments were done on the S-MoS_2 films and an angle of 118 ° was found. These results confirm the hydrophilic surface of M-MoS_2 and hydrophobic surface of S-MoS_2

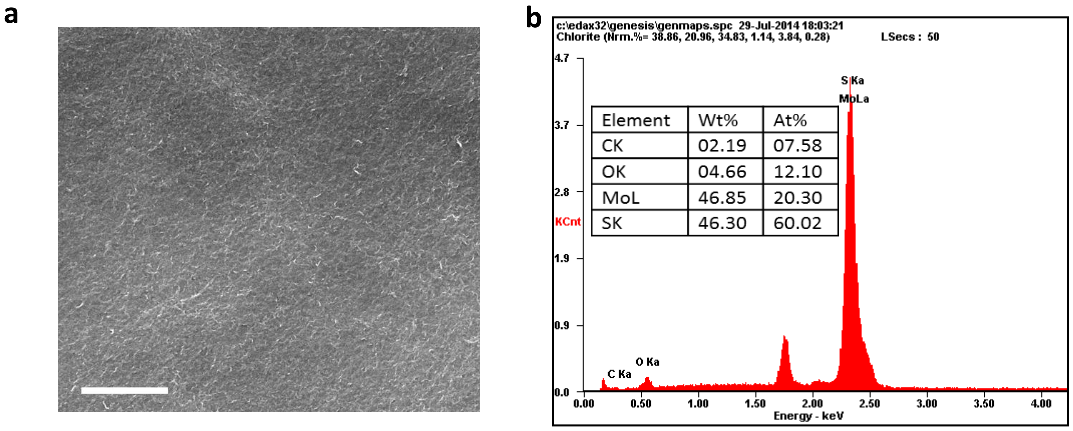

Supplementary Figure 8: Characterization of MoS_3. (a) SEM image of amorphous MoS_3. Scale bar, 1 µm. (b) EDX of MoS_3.

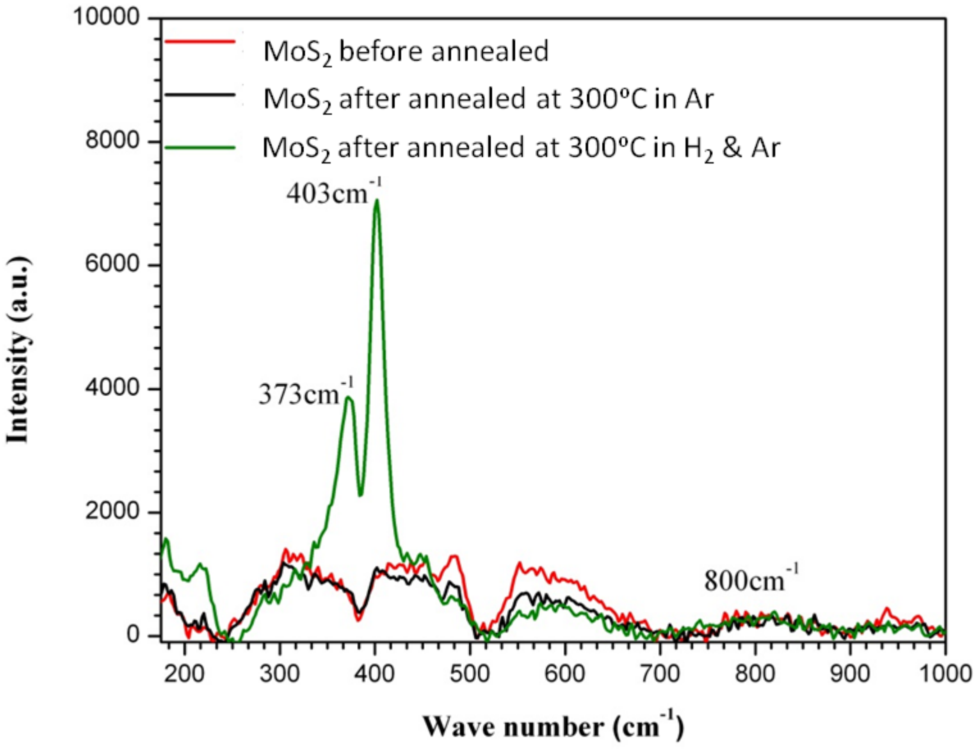

Supplementary Figure 9: Raman shift of MoS_2 before and after annealing in Ar and Ar/H_2 mixture. MoS_2 before annealing (Red), MoS_2 after annealing at 300 ^oC in Ar (Black), MoS_2 after annealing at 300 ^oC in H_2 and Ar (Green),

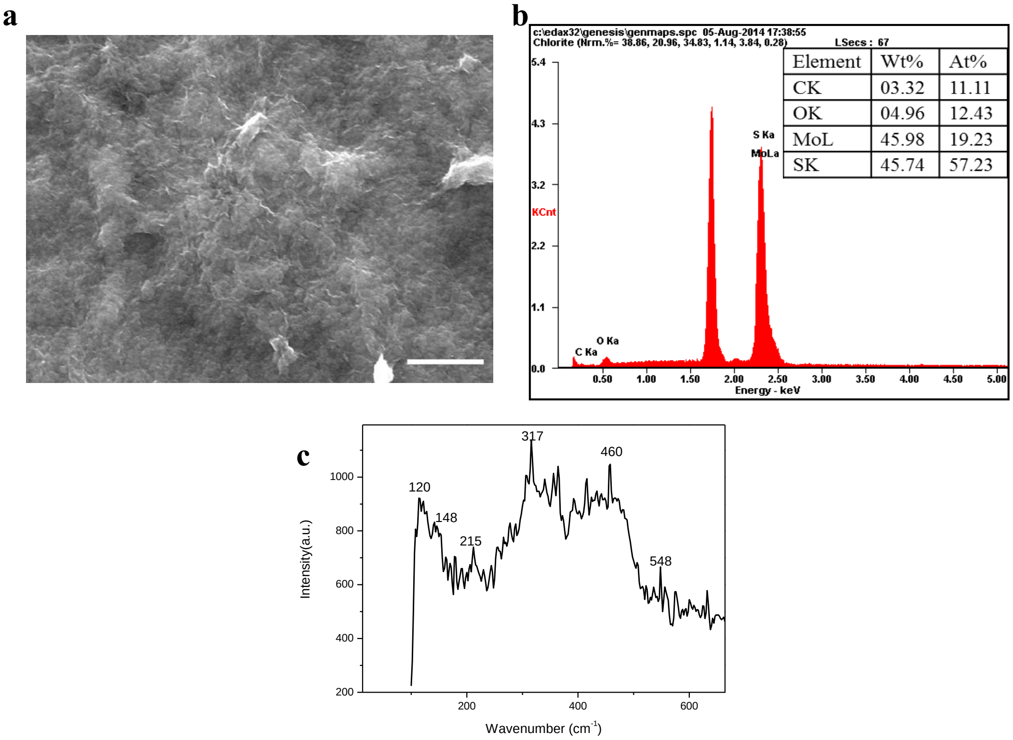

Supplementary Figure 10: Characterization of as-prepared intermediate phase MoS_3. (a) SEM image of amorphous intermediate. Scale bar, 1 μm. (b) EDX result of intermediate and element ratio above. (c) Raman spectrum for samples prepared for one-and-a-half hours. The brown-black solution of MoS_3 showed peaks at 120 cm^{-1}, 215, 317, 460 cm^{-1} in Raman spectra. Meanwhile, a weak metal-metal peak located at 146 cm^{-1} was detected. Only a metal-metal Raman peak at 146 cm^{-1} was observed if the reaction was stopped after six hours (see Figure 1b).

Supplementary Note 1

XPS of M-MoS_2

De-convolution of these peaks in XPS of M-MoS_2 reveals that the ratio of M-MoS_2 to S-MoS_2 is close to 6:1 (see Supplementary Figure 3). As a result, the M-MoS_2 content is estimated to be ~ 86% in the sample. However, this value does not reflect the content of the freshly prepared M-MoS_2. The XPS was conducted at high vacuum for a few hours, during which some of the M-MoS_2 converts to S-MoS_2. This phase transformation in vacuum was confirmed in our Raman measurements. Therefore, the Mo components associated with S-MoS_2 in XPS reflect the phase transformation of M-MoS_2 to S-MoS_2 in the vacuum chamber.

Supplementary Discussion

Exchange current density

Exchange current density is the rate of reaction at the reversible potential. At the reversible potential, the reaction is in equilibrium, meaning that the forward and reverse reactions progress at the same rates. We have used the linear part of the polarization curve at small over-potentials in H_2-saturated 0.5 M H_2SO_4 solution to obtain j_0 values. The exchange current density can be calculated by equation below.

$$\frac{\Delta j}{\Delta \eta} = j_0(\mathrm{nF/RT})$$

Here n represents the number of electrons exchanged, F (96485 C mol^{-1}) is the Faraday constant, and R (8.314 J $mol^{-1}K^{-1}$) is the gas constant. The exchange current densities of 0.55, 0.1 and 0.04 mA cm^{-2} were obtained for Pt, M-MoS_2 and S-MoS_2 from the polarization curves.

Supplementary Methods

Preparation of MoS_3

12 mg MoO_3 and 14 mg thioacetamide were dissolved in 10 ml deionized water and stirred for two hours. The solution was added to the autoclave and then loaded into the furnace at 200 ^oC for twelve hours. After that, the reaction was stopped by rapidly cooling the solution down to room temperature. The yielded MoS_3 was collected and washed in deionized water several times. The as-prepared MoS_3 is amorphous structure as shown by SEM and the ratio of Mo to S is 1:3 as measured by EDX (Supplementary Figure 8).

Prepare S-MoS_2 by different procedure

12 mg MoO_3, 14 mg thioacetamide and 0.12 g urea were dissolved in 10 ml ethanol or dimethylformamide (DMF) and stirred for two hours. The solution was added to the autoclave and then loaded into the furnace at 200 ^oC for twelve hours. After that, the reaction was stopped by rapidly cooling the solution down to room temperature. The yielded small amount of precursor of S-MoS_2 was collected and washed in deionized water for several times. Then S-MoS_2 was obtained after the precursor was annealed at 300 ^oC under hydrogen flow of 50 sccm for one hour. Raman shifts show the highly crystalline structure of pure S-MoS_2 in Supplementary Figure 9. It was noticed that hydrogen plays an important role in reducing the precursor to S-MoS_2 since we found that MoS_2 is not produced if only Argon was used during annealing. If the solvent is a mixture of ethanol and water in a 1:1 ratio, no solid product was found in the autoclave, indicating that the type of solvent is important in the formation of MoS_2.

Control experiment of M-MoS_2

12 mg MoO_3, 14 mg thioacetamide and 0.12 g urea were dissolved in 10 ml deionized water and stirred for two hours. The solution was added to the autoclave and then loaded into furnace at 200 ^oC for one-and-a-half and six hours, respectively. After that, the reaction was stopped by rapidly cooling the solution down to room temperature. The yielded solid materials were collected and the ratio of Mo to S was found to be close to 1:3 when the reaction was stopped after one-and-a-half hours. The amorphous structure was characterized and shown in Supplementary Figure 10.
